# Fibrin glue as a protective tool for microanastomoses in limb reconstructive surgery

**DOI:** 10.3205/iprs000073

**Published:** 2015-12-15

**Authors:** Stefan Langer, Thomas A. Schildhauer, Marcel Dudda, Jeannine Sauber, Nick Spindler

**Affiliations:** 1 Department of Plastic, Esthetic and Special Hand Surgery, University Hospital Leipzig, Germany; 2 Department of Trauma, University Hospital Bergmannsheil, Ruhr University Bochum, Germany

**Keywords:** fibrin glue, microanastomosis, free flap, nerve reconstruction, microsurgery

## Abstract

**Aim:** Fibrin glue becomes a more and more routinely used tool for stabilization of microanastomoses and nerve repair. This paper summarizes the technical properties and advantages of its use in a wide variety of microsurgical contexts, and includes an exemplary limb reconstructive case.

**Patients and methods:** A total of 131 patients who had undergone elective and emergency microsurgery mainly of the limbs were retrospectively analyzed, as was the use of free flaps.

**Results:** The use of fibrin glue allows for proper positioning of anastomoses and repaired nerves. No torsion of the pedicle could be seen. The flap survival rated >94%. The fibrin glue could stay in place in >99%. In the rare case of revision, the fibrin glue could easily be removed without damaging the region of the microanastomosis.

**Conclusion:** Fibrin glue should not be used to repair insufficient, i.e., leaking anastomoses, but it does protect the site of anastomosis from tissue and fluid pressure. It prevents the pedickle from torsion and its use facilitates relocation of the microanastomoses in cases of revision surgery.

## Zusammenfassung

**Einleitung:** Fibrinkleber ist ein immer häufiger in der klinischen Routine eingesetztes Tool zur Stabilisierung von Mikroanastomosen und Nervenreparatur.

Dieser Artikel fasst die technischen Eigenschaften und Vorteile im Gebrauch sowie die variable Anwendungsmöglichkeit von Fibrinkleber im mikrochirurgischen Kontext zusammen und illustriert dies an einem extremitätenrekonstruierenden Fallbeispiel.

**Patienten und Methoden:** 131 Patienten, an welchen sowohl elektiv als auch in einer Notfallsituation eine mikrochirurgische Intervention durchgeführt wurde, hauptsächlich in der Extremitäten erhaltenden Chirurgie, wurden retrospektiv untersucht; ebenso die Patienten, welche eine freie Lappenplastik erhalten hatten.

**Ergebnisse:** Die Verwendung von Fibrinkleber in der Mikrochirurgie erlaubt ein exaktes Positionieren der Anastomose von Gefäßen und Nerven. Ein Verdrehen oder Kinking des Pedickels konnte in keinem Fall festgestellt werden. Die Überlebensrate der Lappen betrug >94%. In 99% der Fälle konnte der Fibrinkleber belassen werden. In dem seltenen Fall der Revision konnte der Fibrinkleber leicht entfernt werden ohne Schaden an der Anastomose zu hinterlassen.

**Zusammenfassung:** Fibrinkleber ist nicht geeignet Anastomoseninsuffizienzen oder leckende Anastomosen zu behandeln, allerdings schützt er die Anastomose vor schadendem Druck ausgelöst durch Gewebe oder Flüssigkeit. Er bewahrt den Gefäßstiel vor Verdrehung und hilft dem Operateur beim Wiederauffinden der Anastomose im Fall einer Revision.

## Introduction

After completion of the microvascular anastomosis and onset of reperfusion, the vein is filled with blood and the artery shows its typical pattern of physiological pulsation and kinking. Every microsurgeon suffers from this experience: in some cases, they find the kinking alarming, and the moving artery does not stay at the site desired by the surgeon. Moreover, the tight vein filled with venous blood calls for an outer shield. Microsurgeons have adopted many technical innovations to improve flap survival, e.g., flap design, reduced time of ischemia, post-surgical management, etc. However, at the time the anastomosis is performed, many microsurgeons are uneasy leaving the microsurgical site without sealing and covering this fragile piece of work. The Breast Center in Duesseldorf, Germany, pioneered the use of fibrin glue in supporting microvascular anastomoses. To date, the Duesseldorf team has sealed microvascular anastomoses in over 1,500 free DIEP flaps [1], [2], [3], [4]. The fibrin glue plug is one major factor contributing to a very low complication rate and flap losses of less than 1% [4]. The use of a fibrin sealant which softly encases microvessels without constricting their physiological movements seems to be one key step in the optimal outcome of transplanted tissues. Fibrin sealants are approved as medical products and are commonly used in many surgical fields, such as endoscopic visceral surgery [5], skin grafts [6], and breast surgery [7], [8]; however there are about no levels of evidence supporting its use [9]. Thus, the purpose of this paper is to present a summary of our experience in using fibrin glue as a technical adjunct in a wide variety of microvascular surgery and nerve repair, with a focus on limb reconstruction.

## Patients and methods

In a working period of 18 months over a period of 4 years (2008–2012), all microvascular cases in which fibrin glue was used were recorded. These operations were all performed or supervised by a single surgeon (SL). Data were retrospectively analyzed and ethics committee approval from the University of Leipzig (*AZ 335-13-1811, November 26^th^ 2013*) was obtained. One hundred thirty-one patients (76 male, 55 female) were treated mainly for trauma (53%), infection (27%) and oncological problems (22%). Patient characteristics are shown in Table 1 (Tab. 1).

## Technique

Our standardized protocol for microsurgical procedures was followed. After the onset of reperfusion, arteries and veins were checked for leakage, and if present, additional suturing was performed. The flap was checked for capillary refill and Doppler signals of the pedicle or perforator were recorded. While still under the surgical microscope, the surgeon set the pedicle in place using micro-pickup forceps.

**Figure 1:**
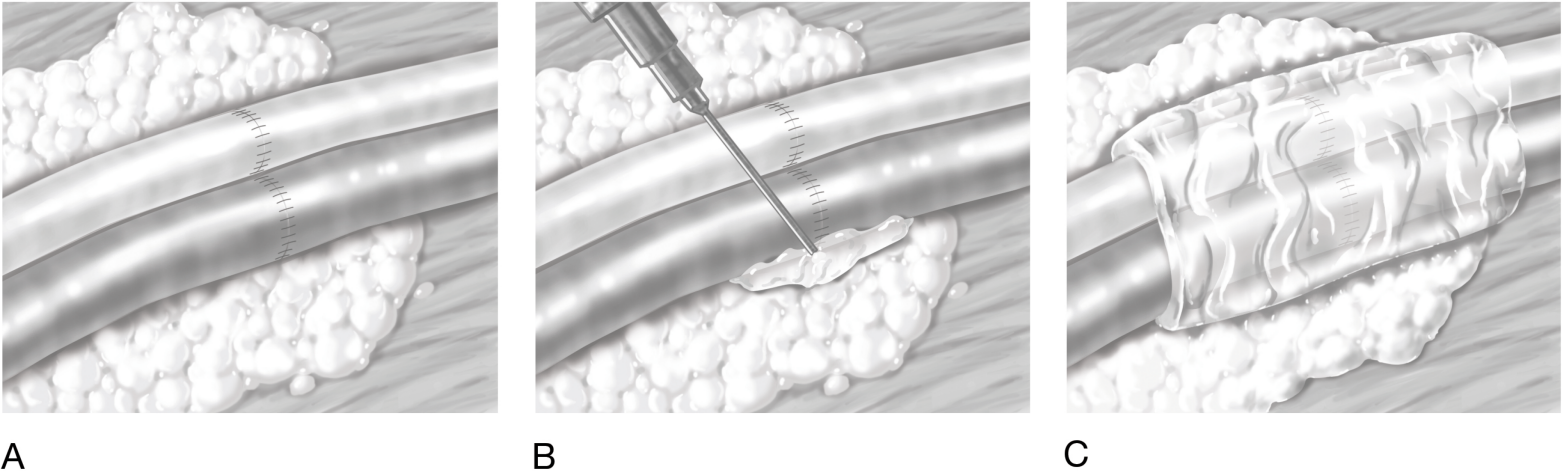
Fibrin glue dual application syringe (www.medillu-camici.de)

Two syringes, containing thrombin and fibrinogen, were joined together within the dual application syringe (Figure 1 (Fig. 1)). Then the assistant surgeon applied the first 0.5 ml of fibrin glue from the application syringe (Tissuecol, Baxter, Vienna, Austria). An average of 1.1 ml of fibrin glue was used in 253 applications in 131 individual patients. The fibrin glue was not applied drop-wise onto the pedicle, but instead the pedicle’s bed was filled from the bottom up until the vessels were completely enveloped within the fibrin clot. Prior to its application, all bleeding, e.g., from the surrounding fat, was stopped. The fibrin glue was applied and the pedicle’s fat pads were placed onto the pedicle for additional protection (Figure 1 (Fig. 1)). Upon completion of these steps, the anastomosis was definitive and the microscope was moved aside. 

## Results

Fibrin glue was used in a wide spectrum of microvascular surgery. End-to-end as well as end-to-side microvascular anastomoses profit from stabilization in terms of less movement with fibrin glue.

No adverse effects were observed during the application (e.g., vasoconstriction of vessels, adverse reaction of surrounded tissues). Moreover, in the rare cases (1%) in which the fibrin glue needed to be removed – redo of leaky anastomoses for instance, no adverse reactions were found. During fibrin glue application by the assistant surgeon, the surgeon is able to definitively position the structures, but after some seconds, the clot is final. However, in a rare cases of anastomotic revision the fibrin clot can easily be removed without any damage to the vessels. This was the case in some emergencies where the surgeon on duty (not familiar with the case) started revision and the operator was called into the OR. Thus, the fibrin clot protects the microvessels from fluid suction and makes the relevant site evident to the surgeon. 

The flap survival rate was good. There were 4 cases of flap failure in peripheral arterial vascular disease patients who had undergone lower limb reconstruction.

### Case report 

**Figure 2:**
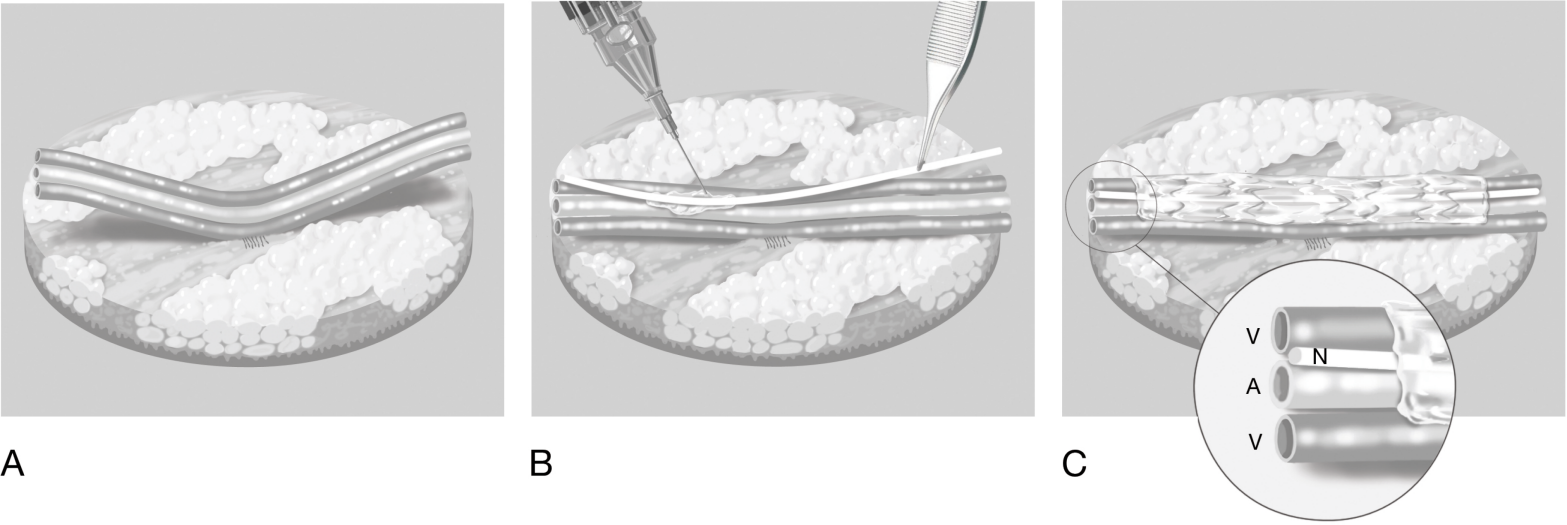
The position of vessels and nerve fixed by the use of fibrin glue (www.medillu-camici.de)

A traumatic ulnar wrist injury needed coverage and neural repair. We decided to use a flow-through ALT flap for reconstruction of a 10 cm-section of nerve, accessory artery and vein. After harvesting the flap, the sural nerve transplant was easily positioned next to the vessels and fixed with glue; no sutures were used. In this way, all structures remain stable during rapid microsurgery (Figure 2 (Fig. 2)).

## Discussion

Today, microsurgical reconstruction has become a valuable, standard operation in our treatment repertoire, and patients requiring or demanding such surgery expect it to be of the highest quality. Thus, surgeons and microsurgeons in particular are always searching for the optimal microsurgical technique to improve daily surgery. In this context, all adjuvant materials and methods that help to achieve optimal results are welcome; for instance, improvements in flap design, surgical instruments, heparin management, as well as anesthesia have furthered this goal. 

After performing the anastomoses of a flap harvested under the best possible conditions and surgeon’s expertise, each individual flap demands optimal follow-up treatment after reperfusion has commenced. Because the consequences of a lost flap are devastating, surgeons continually search for additional adjuvants to improve outcome.

Fibrin glue is widely used in many applications in all surgical fields. Usually, tissue surfaces are the targeted area of application, since fibrin glue reduces hemorrhaging [10], [11] and seroma formation [12], [13]. The use of fibrin glue in facial surgery showed reduction of drainage volume and edema, and thus increased patient satisfaction. It works by reducing a tissue’s dead space, lowering subcutaneous tissue layers in these cases. The use of fibrin glue reduces the numbers of drains placed into surgical wounds as well as their total number of days in situ [14], [15]. 

In spleen and liver surgery, fibrin glue helps to manage hemorrhaging from a parenchymal organ. However, in microvasular surgery, it is not used to staunch bleeding. An insufficient anastomosis should not be covered with fibrin glue; rather, microsurgery should be redone. However, experimental data showed that by use of fibrin glue, an only four-suture anastomosis was sufficient for flap reperfusion [16]. The best time to seal anastomoses is once reperfusion is in progress and outflow drainage is sufficient. 

In breast reconstruction, fibrin glue is routinely used. It helped to reduce flap losses to less than 1% at one center with over 1,500 DIEP flaps [1], [2]. This excellent result is associated with stabilization of the pedicle during the shaping of the breast and its intermittent motion into and out of the breast’s pocket. Thus, the use of fibrin sealing has become an indispensible part of the standard protocol of many breast reconstruction centers. In the rare case that after being fully informed about the risks and benefits of the surgical procedure (informed consent process) a patient nevertheless refuses application of a product derived from human blood, she will not be operated on by this team. 

In microvascular surgery other than breast, the use of fibrin glue provides several advantages. Through its stabilization of the outer vessels walls, the fibrin sealant keeps the vessels from kinking and obviates pressure from hematomas or seromas or, a very important issue in limb reconstruction, from the flaps weight per se. 

The fibrin clot shows the surgeon the site of anastomosis, should problems occur and revision surgery be necessary, which is particularly useful when a surgeon unfamiliar with the case is on call. The guidepost of a fibrin clot helps in our own revision surgery and allows the surgeon to reach the site of the microvascular problem much more quickly and finally save some of the flaps. Once the site of the anastomosis is found, the fibrin clot can easiy be removed by pickup forceps and suctioned off. If the problem is other than the anastomosis, then the problem is solved and the flap is placed back into position. Another advantage is protecting the fragile pedicle from disturbance by a plastic drain; we have observed flap losses due to pedicle torsion after removing a drain. 

There are other methods to protect a vascular pedicle, e.g., placing adipose tissue fragments or muscle beside it [17]. This technique of cushioning tissues can be very efficaciously combined with the use of fibrin glue. Fibrin glue is an excellent tool for filling deep spaces, e.g., at the site of the internal mammaria artery (IMA) or tibial posterior artery anastomoses. As microsurgeons know from breast reconstruction using the IMA, placing the microvessels along a rib is often necessary. To prevent the rib from disturbing the IMA as well as, fibrin glue is used as a distance-maintaining cushion.

## Conclusions

Since compression and kinking of vascular pedicles are associated with disturbed inflow and/or outflow, some surgeons advocate the local application of fibrin glue once the anastomosis is completed, even though fibrin glue is of course not seen as an alternative to sufficient suturing [18]. However, fibrin glue seals small holes within the vessel wall and gives the pedicle some rigidity and adherence to the adjacent tissues, protecting it from shear stress-induced movements while tailoring the flap during its placement or during patients’ movement.

The use of fibrin glue for the stabilization of anastomosed vessels as well as the covering of veins or venous transplants has an important influence on the outcome of microsurgical work [19]. Especially fragile veins profit from coverage with a thin, maleaable and soft material. Fibrin glue may be used as a standard procedure in all kinds of vessel or nerve-based microsurgery, since it provides the optimal feature of controlling microanastomoses.

## Notes

### Acknowledgements

The retrospective study was funded by a research grant from Baxter, Vienna Austria. Fibrin glue was not provided by the company. Medical illustrations were performed by medillu-camici.de. The authors declare that they do not have conflicts of interests concerning the products used in this article.

## Figures and Tables

**Table 1:**
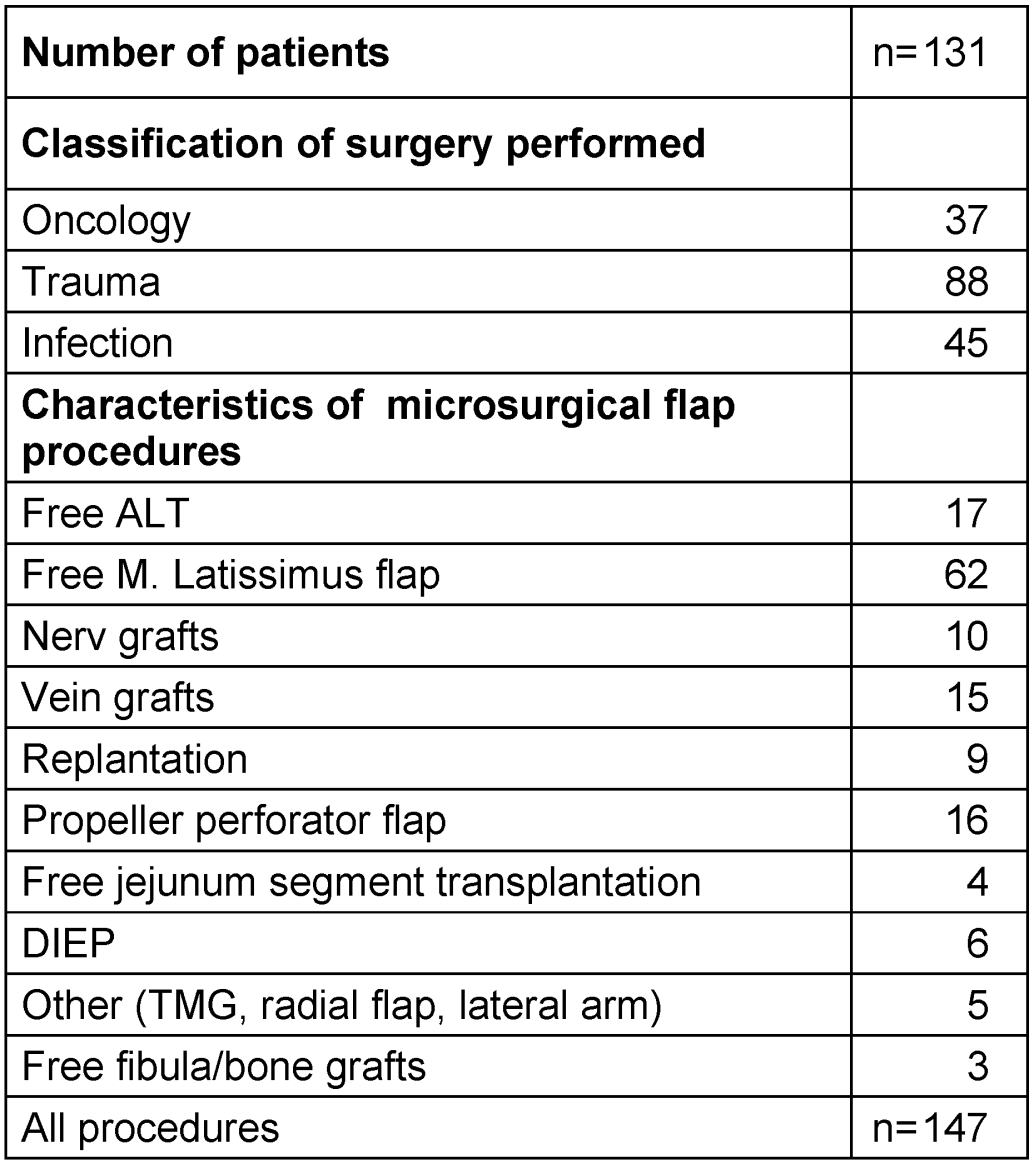
Patient characteristics

## References

[1] Andree C, Langer S, Seidenstuecker K, Richrath P, Behrendt P, Koeppe T, Hagouan M, Witzel C, Al Benna S, Munder B (2013). A single center prospective study of bilateral breast reconstruction with free abdominal flaps: a critical analyses of 144 patients. Med Sci Monit.

[2] Langer S, Munder B, Seidenstuecker K, Richrath P, Behrendt P, Kneser U, Horch RE, Andrews BT, Andree C (2010). Development of a surgical algorithm and optimized management of complications - based on a review of 706 abdominal free flaps for breast reconstruction. Med Sci Monit.

[3] Seidenstuecker K, Munder B, Mahajan AL, Richrath P, Behrendt P, Andree C (2011). Morbidity of microsurgical breast reconstruction in patients with comorbid conditions. Plast Reconstr Surg.

[4] Andree C, Munder BI, Behrendt P, Hellmann S, Audretsch W, Voigt M, Reis C, Beckmann MW, Horch RE, Bach AD (2008). Improved safety of autologous breast reconstruction surgery by stabilisation of microsurgical vessel anastomoses using fibrin sealant in 349 free DIEP or fascia-muscle-sparing (fms)-TRAM flaps: a two-centre study. Breast.

[5] Silecchia G, Boru CE, Mouiel J, Rossi M, Anselmino M, Morino M, Toppino M, Gaspari A, Gentileschi P, Tacchino R, Basso N (2008). The use of fibrin sealant to prevent major complications following laparoscopic gastric bypass: results of a multicenter, randomized trial. Surg Endosc.

[6] Currie LJ, Sharpe JR, Martin R (2001). The use of fibrin glue in skin grafts and tissue-engineered skin replacements: a review. Plast Reconstr Surg.

[7] Sajid MS, Hutson K, Kalra L, Bonomi R (2012). The role of fibrin glue instillation under skin flaps in the prevention of seroma formation and related morbidities following breast and axillary surgery for breast cancer: a meta-analysis. J Surg Oncol.

[8] Miri Bonjar MR, Maghsoudi H, Samnia R, Saleh P, Parsafar F (2012). Efficacy of fibrin glue on seroma formation after breast surgery. Int J Breast Cancer.

[9] Pratt GF, Rozen WM, Westwood A, Hancock A, Chubb D, Ashton MW, Whitaker IS (2012). Technology-assisted and sutureless microvascular anastomoses: evidence for current techniques. Microsurgery.

[10] Ding H, Yuan JQ, Zhou JH, Zheng XY, Ye P, Mao C, Chen Q (2013). Systematic review and meta-analysis of application of fibrin sealant after liver resection. Curr Med Res Opin.

[11] Lochan R, Ansari I, Coates R, Robinson SM, White SA (2013). Methods of haemostasis during liver resection--a UK national survey. Dig Surg.

[12] Bailey SH, Oni G, Guevara R, Wong C, Saint-Cyr M (2012). Latissimus dorsi donor-site morbidity: the combination of quilting and fibrin sealant reduce length of drain placement and seroma rate. Ann Plast Surg.

[13] Sajid MS, Betal D, Akhter N, Rapisarda IF, Bonomi R (2011). Prevention of postoperative seroma-related morbidity by quilting of latissimus dorsi flap donor site: a systematic review. Clin Breast Cancer.

[14] Kamer FM, Nguyen DB (2007). Experience with fibrin glue in rhytidectomy. Plast Reconstr Surg.

[15] Zoumalan R, Rizk SS (2008). Hematoma rates in drainless deep-plane face-lift surgery with and without the use of fibrin glue. Arch Facial Plast Surg.

[16] Han SK, Kim SW, Kim WK (1998). Microvascular anastomosis with minimal suture and fibrin glue: experimental and clinical study. Microsurgery.

[17] Fehm NP, Vatankhah B, Dittmar MS, Tevetoglu Y, Retzl G, Horn M (2005). Closing microvascular lesions with fibrin sealant-attached muscle pads. Microsurgery.

[18] Mücke T, Wolff KD (2009). Performing microvascular anastomosis with fibrin glue--faster, easier, and more reliable?. Microsurgery.

[19] Schwabegger AH, Engelhardt TO, Jeschke J (2008). Stabilization of microvascular pedicles in intricate locations using fibrin glue. Microsurgery.

